# Nurses’ knowledge and attitudes regarding malnutrition in children and its management in Ghana

**DOI:** 10.4102/curationis.v40i1.1618

**Published:** 2017-10-31

**Authors:** Victor Mogre, Alaru Yakubu, Musah Fuseini, Anthony Amalba, Sixtus Aguree

**Affiliations:** 1 Department of Health Professions Education and Innovative Learning, University for Development Studies, Tamale, Ghana; 2 Department of Nursing, University for Development Studies, Tamale, Ghana; 3 Department of Community Nutrition, University for Development Studies, Tamale, Ghana

## Abstract

**Background:**

Malnutrition contributes significantly to child morbidity and mortality. Nurses require appropriate knowledge, skills and attitudes to prevent and treat malnutrition in children using appropriate guidelines or protocols.

**Objectives:**

The aim of this article was to assess nurses’ knowledge, attitudes towards malnutrition and its management using the World Health Organization (WHO) or United Nations International Children’s Fund guidelines for the treatment of severely malnourished children and to evaluate factors associated with their knowledge and attitudes.

**Methods:**

Participants included 104 nurses working in the outpatient and paediatric units or departments of four hospitals in Tamale metropolis. An 88-item questionnaire was used to measure nurses’ socio-demographic characteristics as well as their knowledge and attitudes towards malnutrition in children and its management using the WHO guidelines for the inpatient treatment of severely malnourished children.

**Results:**

Nurses’ knowledge in malnutrition and its management was slightly above average (54.0%), but their attitudes were highly positive. Factors that were associated with nurses’ knowledge were number of nutrition courses undertaken in nursing school, number of years working as a nurse, receipt of a refresher course on nutrition after school and receipt of training on the guidelines. Nurses’ attitudes were associated with report of having awareness on the guidelines, number of years a nurse has been involved in the treatment of a severely malnourished child.

**Conclusion:**

Nurses’ knowledge levels in the inpatient treatment of severely malnourished children were not desirable. However, their attitudes were generally positive. Receipt of previous training, awareness of the WHO guidelines, practice experience and number of years as a nurse significantly affected knowledge and attitude scores in the positive direction.

## Background

Stunting, wasting and other forms of undernutrition contribute significantly to childhood mortality, disease and long-term disability to survivors (Ainsworth 2010; United Nations Children’s Fund 2013). In addition, deficiencies relating to micronutrients such as vitamin A, iron and zinc increase the risk of morbidity and mortality in children. Apart from the immediate devastating effects of undernutrition on morbidity and mortality, undernutrition also causes delayed development affecting children’s cognitive development and outcomes and their productive capacity as adults (Ainsworth 2010).

In 2011, 26% (165 million) of children under the age of five were stunted (low length or height for age) globally (United Nations Children’s Fund 2013). Three fourths of these children live in sub-Saharan Africa and Asia. Approximately 101 million children under the age of five were underweight (low-weight-for-age) in 2011 with about 21% of these children living in sub-Saharan Africa (United Nations Children’s Fund 2013). One in seven Ghanaian children under the age of five was moderately or severely underweight; 23% moderately or severely stunted and 6% moderately or severely wasted (Ghana Statistical Service 2011).

Some of these children become severely malnourished and are admitted to the hospital. Two million children were admitted for severe acute malnutrition (SAM) worldwide in 2011, with over 80% of them coming from sub-Saharan Africa (United Nations Children’s Fund 2013). The risk of death is nine times more likely in malnourished children with or without complications than in well-malnourished children (Black et al. 2008).

Although prevention of SAM is paramount, its occurrence represents a medical emergency and requires urgent and special action to minimise complications and avoid the risk of death (Ashworth et al. 2003).

Case fatality rates for the treatment of severe malnutrition in poor-resourced health centres have been reported to be at 20% – 30% for marasmus (wasting malnutrition) and about 50% – 60% for kwashiorkor for the past 50 years (Collins et al. 2006; Schofield & Ashworth 1996).

Since the 1970s, treatment and management protocols have been introduced and shown to have a capability of reducing case fatalities to as low as 1% – 5% (Ahmed et al. 1999; Golden 1988). One of such protocols is the World Health Organization or United Nations International Children’s Fund (WHO or UNICEF) guidelines for the inpatient treatment of severely malnourished children. The use of these guidelines by hospitals has resulted in a substantial reduction in mortality rates from 30% – 50% to 5% – 15% (Ashworth et al. 2003).

Notwithstanding the positive outcomes of these guidelines as well as other protocols or guidelines in specialised units, their publication has not led to widespread decreases in case fatality rates in most hospitals in developing countries (Briend 2001; Waterlow 2000). Inappropriate case management resulting from poor knowledge (Schofield & Ashworth 1996, 1997) has been attributed to the persistent high case fatality rates in developing countries. Evidence available indicates that the success and effectiveness of the guidelines depend on the availability of skilled and motivated health care staff (Brewster & Manary 1995).

In a country in which malnutrition in children is prevalent, it is very likely that nurses will encounter severely malnourished children in their daily practice. This is even more likely in a health system in which there are few nutritionists or dieticians responsible for managing these conditions. Consequently, the daily routine care for the severely malnourished child as well as education for parents or caregivers is the responsibility of the bedside nurse. Given the role of nurses in paediatric care, they require specialised knowledge and favourable attitudes regarding the treatment of severely malnourished children.

Thus the bridging of knowledge and attitude gaps among nurses is a relevant step towards the development and implementation of targeted educational interventions and ultimate improvement of care provided to inpatient malnourished children.

A number of studies from sub-Saharan Africa (Buxton & Davies 2013; Kgaphola, Wodarski & Garrison 1997; Kobe 2006) and elsewhere (Al-Shwaiyat et al. 2013; Bauer, Halfens & Lohrmann 2015; Boaz et al. 2013; Endevelt et al. 2009; Kim & Choue 2009) have investigated nurses’ knowledge and attitudes towards nutrition. These studies have found nurses to have positive attitudes towards nutrition but limited nutrition-related knowledge.

All of these studies have either investigated nurses’ knowledge and attitudes towards nutrition in general, or considered hospital malnutrition or nutrition of the elderly. Studies investigating nurses’ knowledge and attitudes towards malnutrition in children and its management are limited.

In our search of the literature, we came across only two studies, one from southern Ethiopia (Tafese & Shele 2015) and the other from Guatemala (Hammond 2014) that investigated health workers’ (with nurses inclusive) knowledge, attitude and practice towards malnutrition in children. The authors of both studies found health workers to have inadequate knowledge and skills in clinical nutrition topics although a large proportion of them had positive attitudes towards nutrition issues. Inadequate nutrition education during nursing training and lack of refresher training programmes have been noted for nurses’ poor knowledge in malnutrition.

In Ghana, the situation is not different. Only two studies have investigated nurses’ knowledge and attitudes towards nutrition (Buxton & Davies 2013; Mogre et al. 2015). The study by Buxton et al. (2013) investigated student nurses’ knowledge in nutrition only and found over 30% of the participants having inadequate knowledge. The study by Mogre et al. (2015) evaluated nurses’ knowledge in the nutritional management of diabetes and also found the nurses to have poor knowledge but their attitudes were positive. Although malnutrition is common in Ghana, the evaluation of nurses’ knowledge and attitudes towards malnutrition and its management has not been previously carried out in Ghana.

### Aims of the study

This study assessed nurses’ knowledge and attitudes regarding malnutrition in children and its management using the WHO or UNICEF guidelines for the inpatient treatment of severely malnourished children. Furthermore, factors associated with nurses’ knowledge and attitudes regarding malnutrition in children and its management using the WHO or UNICEF guidelines were also determined.

## The WHO guidelines for the inpatient treatment of severely malnourished children

The WHO guidelines are simple practical guidelines for treating severely malnourished children intended for doctors, nurses, dieticians and other health workers with the responsibility for the medical and dietary management of severely malnourished children, and for their trainers and supervisors. The guidelines are divided into two major phases: the initial stabilisation phase and the rehabilitation phase. Its initial stabilisation phase emphasises on frequent feeding day and night, rehydrating with low-sodium fluids with close monitoring for signs of fluid overload, correcting electrolyte and micronutrient deficiencies and prescribing broad-spectrum antibiotics even when signs of infection are absent (Karaolis et al. 2007). The focus of the rehabilitation phase is the rebuilding of lost body tissues, psychosocial stimulation and preparation for discharge and follow-up. In all, the treatment using the guidelines is executed in 10 essential steps.

## Ethical considerations

The study followed the guidelines of the Helsinki Declaration (World Medical Association). Ethical approval for the study was granted by the Research Unit of the Tamale Teaching Hospital. Institution gate keepers also granted permission to gain access into the study area. Using the informed consent form potential participants were assured voluntary participation; were at liberty to withdraw from the study at any stage without penalty; and anonymity and confidentiality of information provided. Each participant signed the informed consent form. All informed consent procedures were approved by the Research Unit of the Tamale Teaching Hospital, Ghana. Hazards to participants were not anticipated and none were recorded during the study.

## Research methods and design

### Design

This cross-sectional study was conducted in four randomly selected hospitals located in the Tamale Metropolis of Ghana during the period January to July 2014.

### Sampling and setting

All hospitals, both private and public, located in the metropolis were listed and four hospitals were selected through the lot. These hospitals included Tamale Teaching Hospital, Tamale Central and West Hospitals and the Seventh Day Adventist Hospital. All of these hospitals provide both inpatient and outpatient treatment for severely malnourished children.

All nurses with a minimum of two years’ working experience were eligible to participate. Also, nurses who were working in the outpatient departments (OPDs) and the children’s wards of the selected hospitals were eligible to participate in the study. All student nurses and those who were awaiting results from the Nurses and Midwives Licensure Examinations were excluded from the study. Also, nursing administrators were excluded for their limited involvement in the clinical management and treatment of patients.

Permission to have access to the hospital was granted by the directors of the hospitals and the respective unit, department or ward heads. Nurses were approached during unit or departmental meetings to introduce the study to them and to seek for their consent to participate in the study. Through this process all nurses working in the OPDs and children wards of the selected hospitals were approached to participate in the study.

One hundred and twenty questionnaires were distributed among those who consented and agreed to participate in the study, from which 104 questionnaires were returned yielding a response rate of 86.7%. Among the questionnaires returned 25 were from the Tamale central hospital, 23 from West hospital, 20 from Seventh Day Adventist hospital and 36 from the teaching hospital.

### Instrumentation

The knowledge and attitude levels in the inpatient treatment of severely malnourished children of the nurses were assessed using an 88-item survey instrument prepared by the authors i.e. author-created. The first fifteen items of the questionnaire assessed nurses’ socio-demographic and professional background data including age and gender. Data on the professional background included the highest level of nursing education, number of years as a nurse, number of nutrition courses taken in school, among others.

The items for the knowledge and attitude scales of the questionnaire were derived from the WHO or UNICEF guidelines for the inpatient management of severely malnourished children, referred to as the guidelines in this article. The knowledge scale consisted of 26 multiple-choice questions and 15 true or false questions. It covered seven content areas of the guidelines: the causes and signs of malnutrition (11 items), phases of the guidelines (4 items), treatment or prevention of hypoglycaemia and hypothermia (10 items), dehydration (6 items), electrolyte imbalance (4 items), infections and correct micronutrient deficiencies (4 items) and achievement of catch-up growth and cautious feeding (2 items). Cronbach’s alpha analysis of this component of the questionnaire indicated a good level of internal consistency (Cronbach’s alpha = 0.83). Each of the 41 items obtained an equal point of 1 if answered correctly yielding a total score of 41 to represent knowledge score. Questions that were not answered correctly were considered as incorrect. Subtotal scores for each of the seven content areas were determined by the number of items that made up the content area.

The attitude scale consisted of 28 items (presented as statements) defined on a five-point Likert scale. Participants were required to determine the extent to which they agreed or disagreed to each of the statements by indicating 1 = totally disagree; 2 = disagree; 3 = neutral; 4 = agree; and 5 = totally agree. For example, to what extent do you agree or disagree to the statement ‘Malnourished children do not respond to medical treatment in the same way as if they were well-nourished?’ Similar to the knowledge part of the questionnaire, these statements were derived from the WHO or UNICEF guidelines. Each item was scored from 1 to 5, to yield a total score of 140 to represent attitude score. High scores indicated positive attitude and low scores suggested poor attitude. Cronbach’s alpha analysis of the attitude component of the questionnaire revealed a satisfactory level of reliability and internal consistency (alpha = 0.69).

Two items were included in the questionnaire to assess nurses’ awareness level and practice of the guidelines. Awareness level was assessed by indicating yes or no to the question, ‘Have you ever heard of the WHO or UNICEF guidelines for the inpatient management of severely malnourished children?’ Nurses’ practice level of the guidelines was assessed by the question, ‘Have you ever been involved in the management of a severely malnourished child? Yes or No’.

The test items were submitted to a panel of three experts in nutrition and health for evaluation of content validity, test format and item construction. With revisions based on comments of the panel, the items were approved by the panel as the content was valid and appropriate. The final questionnaire was piloted on a sample of fifteen nurses to assess the comprehensibility of the items to participants. These nurses did not participate in the study but worked in a similar area. The questionnaire was also reviewed by a panel of nutrition experts who considered the questions to have content validity.

### Data analysis

Descriptive statistics of mean, standard deviation and frequencies were used to describe the data. Student *t*-test was performed to compare differences in knowledge and attitude scores between categorical variables. Nonparametric Spearman rank correlation was used to determine associations between continuous variables. Cronbach’s alpha and total-item correlation were used to determine the internal consistency of the knowledge and attitude scales of the questionnaire.

A *p*-value of < 0.05 was considered statistically significant. IBM SPSS statistics 21.0 was used to perform the Cronbach’s alpha analysis and the nonparametric Spearman rank correlation. The student *t*-test analysis was performed using GraphPad Prism version 5.00 for Windows (GraphPad Software, San Diego California USA, www.graphpad.com).

## Results

Table 1 presents characteristics of the participants. These nurses had the following highest level of education: certificate 32.7% (*n* = 34); diploma 42.3% (*n* = 44); BSc 21.2% (*n* = 22); and postgraduate 3.8% (*n* = 4). Almost all nurses had taken nutrition courses as part of their training in school; 53.8% (*n* = 56) took ≤ 2 courses in nutrition and 46.2% (*n* = 48) > 2 courses. Generally, 91.3% (*n* = 95) of the nurses reported ever being taught on malnutrition in children. Fewer than half (48.1%, *n* = 50) of the nurses reported having awareness on the guidelines. Their source of awareness was also assessed. Almost one half (44.0%, *n* = 22) of the nurses obtained their awareness through reading relevant sites on the Internet. Other sources of information were used sparingly; health information articles were read by 36.0% (*n* = 18) of the nurses, and workshops were attended by 16% (*n* = 8) of the nurses. Only nineteen nurses (18.3%) reported ever been trained on the guidelines, in which 99% of them were trained within the last five years (2010–2014), and only 1 in 2009. Even though nineteen nurses reported ever been trained on the guidelines, 49 (47.1%) of them said they have ever been involved in the treatment of a severely malnourished child. The roles they played included feeding, medication, monitoring of vital signs and nutrition education to parents or caregivers.

**TABLE 1:** General and professional characteristics of the nurses (*n* = 104).

Variable	*n* (%)
Gender	
Male	61 (58.7)
Female	43 (41.3)
Mean age	28.64 ± 5.34
Age category	
≤ 30	82 (78.8)
> 30	22 (21.2)
Mean years as a nurse	4.82 ± 4.92
Mean no. of hours per week for nutrition in school	3.07 ± 1.74
Ever had a refresher course in nutrition after school	
Yes	32 (30.8)
No	72 (69.2)
Has awareness of WHO guidelines for SAM	
Yes	50 (48.1)
No	54 (51.9)
Ever had a training workshop on the management of SAM	
Yes	19 (18.3)
No	85 (81.7)
Ever been involved in the management of a severely malnourished child	
Yes	49 (47.1)
No	55 (52.9)

SAM, severe acute malnutrition.

**FIGURE 1:**
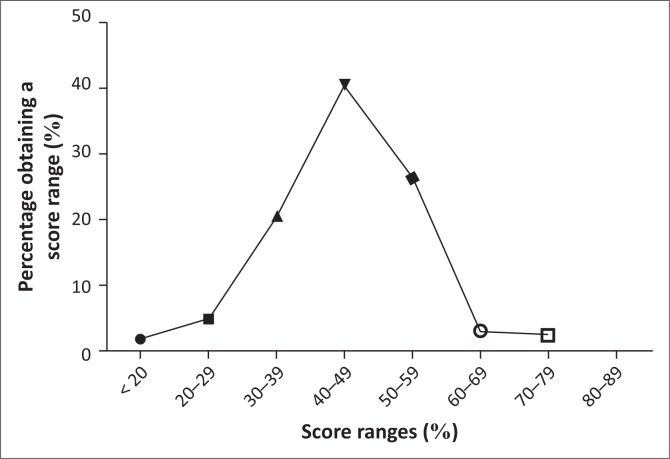
Frequency of distribution of the knowledge scores of the nurses.

The frequency distribution of the scores as shown in Figure 1 indicated a bell-shaped curve. The nurses obtained a mean knowledge score of 54.10 ± 16.61.

The mean knowledge scores of the nurses according to the seven content areas that were assessed are presented in Table 2. The nurses obtained high scores on the questions that addressed the causes and signs of malnutrition but had below average (46.8 ± 23.00) scores for questions that focused on how to treat or prevent hypoglycaemia and hypothermia.

**TABLE 2:** Nurses’ mean knowledge scores according to the seven topics assessed (*n* = 104).

Content	Mean ± SD score
Causes and signs of malnutrition (max. score = 11)	73.73 ± 19.09
Phases of the guidelines (max. score = 4)	35.25 ± 36.50
Treat or prevent hypoglycaemia and hypothermia (max. score = 10)	46.88 ± 23.00
Treat or prevent dehydration (max. score = 6)	49.17 ± 25.17
Correct electrolyte imbalance (max. score = 4)	53.75 ± 25.50
Treat or prevent infections and anaemia (max. score = 4)	45.50 ± 27.25
Cautious feeding and achieve catch-up growth (max. score = 2)	50.00 ± 28.00

SD, standard deviation.

The percentage of correct answers to the individual knowledge items of the questionnaire is presented in Table 3. Almost 63% (*n* = 65) of the nurses were unable to recognise the cardinal signs for kwashiorkor. Regarding feeding frequency to treat or prevent hypoglycaemia in a severely malnourished child, 68.3% (*n* = 33) were unable to correctly indicate that the child should be fed every 30 minutes for 2 hours. Approximately 56% (*n* = 58) of the nurses did not know that feeds should be given throughout the night to treat or prevent hypothermia in a severely malnourished child. About 41% (*n* = 43) of the nurses incorrectly answered that the IV route should be used at all times to treat dehydration in severely malnourished children. Pathetically, 57.7% (*n* = 60) of the nurses incorrectly reported that the standard oral rehydration salts (ORS) should be used for the treatment of dehydration in severely malnourished children. More than 70% (*n* = 74) of the nurses did not know that fever is not often present as a usual sign of infection in severely malnourished children. Fifty-seven percent (*n* = 59) of the nurses did not know that it is not appropriate to prescribe iron to treat anaemia in the initial phase as it could result in the death of the severely malnourished child. Seventy-six percent (*n* = 79) of the nurses could not recognise that it is not appropriate to prescribe a high-protein diet for a severely malnourished child.

**TABLE 3:** Percentage of correct answers to the individual knowledge items.

Questionnaire item	*N* (%)
Malnutrition refers to the imbalance between energy intake and expenditure	86 (82.3)
Undernutrition is the commonest form of malnutrition in Ghana	92 (88.5)
A deficiency of energy in children results in marasmus	78 (75.0)
A deficiency of protein in children results in kwashiorkor	89 (85.6)
Bilateral pitting odema is a cardinal sign of kwashiorkor	39 (37.5)
Severe acute malnutrition is diagnosed by the presence of < 70% weight-for-age or ≤ 3 SD and/or oedema on both feet	48 (46.2)
Knows methods used in diagnosing malnutrition in children	54 (51.9)
Knows the clinical manifestations of severe acute malnutrition	14 (13.5)
Knows the number of essential steps contained in the guidelines	31 (29.8)
Knows the two treatment stabilisation and rehabilitation phases of the guidelines	47 (45.2)
Acute medical conditions are usually managed in the stabilisation phase of the guidelines	29 (27.9)
To treat or prevent hypoglycaemia is the first essential step of the guidelines	40 (38.5)
Hypothermia and hypoglycaemia usually coexist in severely malnourished children	42 (40.4)
Assume every severely malnourished child is hypglycaemic if you are unable to test for blood glucose	57 (54.8)
Frequent feeding is usually needed to treat or prevent hypoglycaemia in severely malnourished children	71 (68.3)
The IV route should be used to rehydrate severely malnourished children who are dehydrated	49 (47.1)
Standard oral rehydration salts should not be used to treat or prevent dehydration in severely malnourished children	31 (29.8)
Diuretics should not be used to treat oedema in severely malnourished children	42 (40.4)
Salt should not be included in foods prepared for severely malnourished children	59 (56.7)
Fever is usually absent in severely malnourished children	19 (18.3)
Iron should not be given to severely malnourished children during the initial phase of treatment	39 (37.5)
Severely malnourished children should be weighed each morning before feeding	86 (82.7)
High-protein diets should not be prescribed for children presenting with kwashiorkor	16 (15.4)

SD, standard deviation; IV, intravenous.

With a mean attitude score of 117.7 ± 15.06 (maximum score = 140), the nurses indicated positive attitudes towards the inpatient management of severely malnourished children as shown in Table 4. Eighty percent(*n* = 83) of the nurses felt that malnourished children do not respond to medical treatment in the same way as well-nourished children. The nurses felt that severe malnutrition represents a medical emergency (82.6%, *n* = 91); the risk of death is higher in malnourished children compared to their well-nourished (85.6%, *n* = 89) counterparts and severely malnourished children need special care (93.2%, *n* = 97). With regard to the nurses’ attitudes towards their responsibility, almost 85% (*n* = 86) of the nurses indicated that they were responsible for the medical and dietary management of malnourished children. Ninety percent (*n* = 94) of the nurses felt that feeding of the malnourished child is the responsibility of the nurse. Furthermore, nurses believed that nutrition is very critical in the treatment of severe malnutrition (89.5%, *n* = 93) and that it was their responsibility to provide nutrition education to the parents or care givers of the malnourished child (73.1%, *n* = 76). The nurses recognised the importance of team work in the provision of care, as 94.3% (*n* = 98) of them agreed that team work is necessary for the management of severe malnutrition. Despite the general positive attitude reported towards the inpatient management of severely malnourished children, a few deficiencies in attitudes were also noted. Most of the nurses did not believe that it was their responsibility to undertake the nutritional assessment of the malnourished child, 78.9% (*n* = 82) of them felt that nutritional assessment of the malnourished child is the sole responsibility of the nutritionist or dietician. As 43.4% of the nurses felt it was their responsibility to assess the nutritional status of the malnourished child, 53.9% of them said it was the responsibility of the doctor. About 52% (*n* = 54) of the nurses indicated that worst outcome should always be expected in severely malnourished children.

**TABLE 4:** Nurses’ attitudes towards malnutrition in children and its management.

Variable	Response		
Attitude statements	Disagree (%)	Neutral (%)	Agree (%)
The medical and dietary management of malnourished children is my responsibility	11 (10.6)	(6.7)	86 (82.7)
The nutritionists and dieticians are responsible for medical and dietary management of malnourished children	67 (64.4)	7 (6.7)	30 (28.9)
Poor nutrition hinders personal, social and national development	7 (6.7)	6 (5.8)	91 (87.5)
With appropriate inpatient and follow-up care, the lives of many children can be saved	2 (1.9)	6 (5.8)	96 (92.3)
All malnourished children have excess body sodium though plasma sodium may be low	12 (11.6)	37 (35.6)	55 (52.8)
In children, infection and malnutrition are related	12 (11.6)	9 (8.7)	83 (81.7)
Severely malnourished children have vitamin and mineral deficiency	14 (13.5)	14 (13.5)	76 (73.0)
There is delayed mental and behavioural development in SAM	5 (4.8)	13 (12.5)	86 (82.7)
Ensure good nutrition to prevent severe malnutrition	4 (3.8)	3 (2.9)	97 (93.3)
In the management of SAM, frequent feeding is important	3 (2.9)	10 (9.6)	91 (87.5)
A malnourished child who is crying excessively should be ignored	67 (64.5)	7 (6.7)	30 (28.8)
Sympathy and empathy are needed to nurse the malnourished child	6 (5.8)	7 (6.7)	91 (87.5)
You feel worse when criticised for your handling of a severely malnourished child	50 (48.1)	30 (28.8)	24 (23.1)

The knowledge and attitude scores of the nurses was stratified by socio-demographic, practice and professional background variables and compared using student’s *t*-test as presented in Table 5. Gender did not have an influence on the knowledge scores of the nurses. Nurses who reported ever receiving a refresher course on nutrition training after school significantly scored higher than those who reported no receipt of a refresher course (25.5 ± 6.55 *v* 20.7 ± 6.82; *p* = 0.001). Receipt of training on the guidelines also influenced the knowledge scores of the nurses. The mean knowledge scores of nurses who reported receipt of the guidelines was 24.68 ± 6.22 compared to the 19.87 ± 6.15 obtained by those who reported no receipt of such training (*p* = 0.004). Attitude scores were significantly influenced by report of having awareness of the guidelines and ever been involved in the treatment of a severely malnourished child.

**TABLE 5:** Factors associated with nurses’ knowledge and attitude scores.

Variable	Knowledge score	Attitude score
Gender		
Male	55.14 ± 15.78	118.10 ± 15.46
Female	52.46 ± 16.63	117.10 ± 14.63
*p*-value	0.3873	0.749
Had a refresher course on nutrition after school		
Yes	62.20 ± 16.98	120.10 ± 15.65
No	50.51 ± 14.93	116.70 ± 14.79
*p*-value	0.001	0.304
Has awareness on the WHO guidelines for SAM		
Yes	60.19 ± 15.17	122.40 ± 14.94
No	48.46 ± 15.00	113.30 ± 13.93
*p*-value	0.001	0.002
Ever been trained on the WHO guidelines for management of SAM		
Yes	65.73 ± 17.36	123.80 ± 17.91
No	51.51 ± 14.71	116.30 ± 14.11
*p*-value	0.004	0.050
Ever been involved in the management of a severely malnourished child in accordance with the guidelines		
Yes	60.88 ± 15.76	121.90 ± 15.72
No	48.29 ± 14.22	113.90 ± 13.51
*p*-value	< 0.001	0.007

As shown in Table 6, a significant positive relationship was observed between nurses’ age, knowledge and attitude scores. As the number of nutrition courses undertaken in school increased, knowledge scores increased. Also the number of years as a nurse was found to correlate positively with knowledge (*r* = 0.302, *p* < 0.001) and attitude scores (*r* = 0.206, *p* < 0.05). Furthermore, as knowledge scores increased, attitude scores increased (*r* = 0.379, *p* < 0.001).

**TABLE 6:** Spearman’s nonparametric correlation of factors associated with knowledge and attitude scores.

Variable	NC	TNC	NYN	KS	AS
Age	−0.004	−0.152	0.781**	0.210*	0.249*
No. of nutrition courses taken in school (NC)	-	0.108	0.051	0.215*	0.105
Time (in hours) spent per week for the nutrition course (TNC)	-	-	−0.135	0.126	0.001
No. of years as a nurse (NYN)	-	-	-	0.302**	0.206*
Knowledge score (KS)	-	-	-	-	0.379**

AS, attitude score.

** Correlation is significant at the 0.01 level (2-tailed); *, Correlation is significant at the 0.05 level (2-tailed).

## Discussion

The current study assessed nurses’ knowledge and attitudes towards malnutrition and its management in children using the WHO guidelines for the inpatient management of severely malnourished children. Factors associated with their knowledge and attitudes were also investigated. Nurses’ knowledge in malnutrition and its management was found to be inadequate. However, nurses had positive attitudes towards malnutrition and its management in children. Factors that were found to be associated with nurses’ knowledge and attitudes were previous training and awareness on the management of malnutrition using the guidelines, number of years as a nurse, number of nutrition courses taken in school and age.

The nurses’ mean knowledge score in this study was found to be 54%, which was slightly above average and could be considered as inadequate in knowledge regarding malnutrition and its management. Similar findings have been reported among health workers in Ethiopia and Guatemala (Hammond 2014; Tafese & Shele 2015).

From the seven content areas assessed in this study, the nurses scored better in the questions that addressed their general knowledge on the causes of malnutrition (shown in Table 2) but scored below average on the questions that addressed the signs and symptoms of the different forms of malnutrition such as kwashiorkor. Important knowledge gaps were also noted regarding areas that focused on the clinical nutritional management of severely malnourished children (see Table 3). For instance, only 15% of the nurses knew that high-protein diets should not be prescribed for children presenting with kwashiorkor. Also, about 70% of them did not know that standard ORS should not be used to treat or prevent dehydration in severely malnourished children (see Table 3). The less desirable knowledge levels of the nurses regarding malnutrition and its management could be due to the inadequacies in nutrition education during nursing training. In this study, nurses said they received an average of two courses (3 hours a week for each course) on nutrition in their entire nursing training in school. Furthermore, only 31% of them reported ever attending a refresher course in nutrition after school and a much smaller percentage of 18% reported receiving training on the WHO guidelines for the inpatient treatment of severely malnourished children. Previous studies from several countries have also pointed to the fact that nutrition training during nursing school is not given the needed priority and has been inadequate (Kobe 2006; McWhirter & Pennington 1994; Waitzberg, Caiaffa & Correia 2001).

Another important finding of this study was that nurses who reported ever receiving a refresher course in nutrition or training on the WHO or UNICEF guidelines had significantly higher scores in knowledge than those who did not (see Table 5). Furthermore, those who said they had awareness on the guidelines also tended to score higher than those who reported lack of awareness (see Table 5). This was made more evident when a positive correlation was observed between the number of nutrition courses taken in nursing school and knowledge scores. This recognises the role of nutrition education during nursing training and also supports the idea of providing refresher training programmes for nurses to help build their capacity in malnutrition and its management.

Clinical experience in the management of severely malnourished children was found to be independently associated with knowledge and attitude scores (see Table 5). Nurses who reported ever participating in providing care to severely malnourished children scored significantly higher in the knowledge and attitude scores than those who did not. Similar to our findings, Kobe (2006) reported that ever conducting nutritional assessments correlated directly with knowledge scores in a study that determined the nutritional knowledge, attitudes and practices of nurses. Similar findings have also been reported by Yacin et al. (2013) in a study that assessed the nutritional knowledge of nurses in Turkey.

In general, the nurses showed positive attitudes towards malnutrition and its management in children. However, some deficiencies were noted especially with regard to the nutritional assessment of the malnourished child. Although the nurses agreed that it was their responsibility to always feed the severely malnourished child, a large proportion of them felt that it was not their responsibility to undertake the nutritional assessment of the malnourished child but the sole responsibility of nutritionists or dieticians (see Table 4). This is concerning because most health institutions in Ghana have few nutritionist or dieticians or have none to provide this care to the severely malnourished child. These nurses may rely on medical doctors, as over half of them felt that it was the responsibility of the doctor to undertake the nutritional assessment of the severely malnourished child in the absence of the nutritionists or dieticians. However, doctors may lack the confidence to provide this care because it has been recognised widely that doctors’ knowledge and skills in nutrition are not adequate (Kushner 1995; Mihalynuk et al. 2008). This may result in the provision of limited care to the severely malnourished child ultimately affecting treatment outcomes.

Given the positive correlation between knowledge and attitude scores in this study as well as the positive influence of previous training on knowledge and attitude scores, nurses’ attitudes could be changed by improving their knowledge and skills in the relevance, essence and the know-how of nutritional assessment and the management of malnutrition. This could be done through continuous education programmes in the form of workshops and short courses on the guidelines.

Another important finding of this study was that age correlated positively with knowledge and attitude scores (see Table 6). Also, number of years as a nurse correlated positively with knowledge and attitude scores (see Table 6). The literature on the influence of demographic factors of age and number of years of experience on the nutrition knowledge of nurses are inconclusive. While several studies have reported findings similar to ours (Park et al. 2011; Schaller & James 2005), others have either found no relationship or the reverse (Al-Shwaiyat et al. 2013; Crogan, Shultz & Massey 2000; Yalcin et al. 2013). Nonetheless, these findings suggest that in designing targeted educational interventions to improve the nutrition knowledge of nurses, consideration should be given to these demographic factors.

## Strengths and limitations

This study tackled a critical gap in the literature i.e. the unavailability of data relating to the knowledge and attitude of nurses regarding malnutrition and its management in children. It provided evidence regarding the inadequacy of nutritional knowledge among nurses. It further brings to bear the inadequacies in nutrition education during nursing training and provides important points for curricula planning and change. The cross-sectional nature of this study makes it difficult to establish causality. The generalisability of the findings of this study is limited by the use of the purposive sampling to select the study participants. The use of a questionnaire to measure attitude increases the likelihood of social desirability biases. However, the questionnaire was self-administered reducing the effect of this bias.

## Conclusion

Nurses’ knowledge in the inpatient management of severely malnourished children was inadequate. However, their attitudes were generally positive. Attitude and knowledge were correlated. Receipt of previous training, awareness of the WHO guidelines and number of years as a nurse significantly affected knowledge and attitude scores in the positive direction. Refresher programmes present an opportunity to improve nurses’ knowledge and attitude in the inpatient treatment of severely malnourished children.

## References

[CIT0001] Ahmed T., Ali M., Ullah M.M., Choudhury I.A., Haque M.E., Salam M.A. et al., 1999, ‘Mortality in severely malnourished children with diarrhoea and use of a standardised management protocol’, *The Lancet* 353(9168), 1919–1922. 10.1016/S0140-6736(98)07499-6 10371570

[CIT0002] Ainsworth M, 2010, *What can we learn from nutrition impact evaluations?: Independent evaluation group studies*, The World Bank, Washington, DC.

[CIT0003] Al-Shwaiyat N.M., Sinjillawi A.B., Al-Rethaiaa A.S., Fahmy A.-E.A., Al-Saraireh R.M., Aqel M.M. et al., 2013, ‘Assessment of therapeutic nutritional knowledge of Jordanian nurses’, *International Journal of Nutrition and Food Sciences* 2(3), 142–148. 10.11648/j.ijnfs.20130203.18

[CIT0004] Ashworth A., Khanum S., Jackson A., Schofield C. & Organization W.H, 2003, *Guidelines for the inpatient treatment of severely malnourished children*, World Health Organization, Geneva.

[CIT0005] Bauer S., Halfens R.J. & Lohrmann C, 2015, ‘Knowledge and attitudes of nursing staff towards malnutrition care in nursing homes: A multicentre cross-sectional study’, *The Journal of Nutrition, Health & Aging* 19(7), 734–740. 10.1007/s12603-015-0535-7 PMC12878964 26193856

[CIT0006] Black R.E., Allen L.H., Bhutta Z.A., Caulfield L.E., De Onis M., Ezzati M. et al., 2008, ‘Maternal and child undernutrition: Global and regional exposures and health consequences’, *The Lancet* 371(9608), 243–260. 10.1016/S0140-6736(07)61690-0 18207566

[CIT0007] Boaz M., Rychani L., Barami K., Houri Z., Yosef R., Siag A. et al., 2013, ‘Nurses and nutrition: A survey of knowledge and attitudes regarding nutrition assessment and care of hospitalized elderly patients’, *The Journal of Continuing Education in Nursing* 44(8), 357–364. 10.3928/00220124-20130603-89 23758072

[CIT0008] Brewster D. & Manary M, 1995, ‘Treatment of severe malnutrition’, *The Lancet* 345(8947), 453 10.1016/S0140-6736(95)90435-2 7741871

[CIT0009] Briend A, 2001, ‘Management of severe malnutrition: Efficacious or effective?’, *Journal of Pediatric Gastroenterology and Nutrition* 32(5), 521–522. 10.1097/00005176-200105000-00005 11429509

[CIT0010] Buxton C. & Davies A, 2013, ‘Nutritional knowledge levels of nursing students in a tertiary institution: Lessons for curriculum planning’, *Nurse Education in Practice* 13(5), 355–360. 10.1016/j.nepr.2012.09.014 23083895

[CIT0011] Collins S., Dent N., Binns P., Bahwere P., Sadler K. & Hallam A, 2006, ‘Management of severe acute malnutrition in children’, *The Lancet* 368(9551), 1992–2000. 10.1016/S0140-6736(06)69443-9 17141707

[CIT0012] Crogan N.L., Shultz J.A. & Massey L.K, 2000, ‘Nutrition knowledge of nurses in long-term care facilities’, *Journal of Continuing Education in Nursing* 32(4), 171–176. 10.3928/0022-0124-20010701-08 11868957

[CIT0013] Endevelt R., Werner P., Goldman D. & Karpati T, 2009, ‘Nurses’ knowledge and attitudes regarding nutrition in the elderly’, *JNHA-The Journal of Nutrition, Health and Aging* 13(6), 485–489. 10.1007/s12603-009-0098-6 PMC12880351 19536416

[CIT0014] Ghana Statistical Service, 2011, *Ghana multiple indicator cluster survey with an enhanced malaria module and biomarker*, Ghana Statistical Service, Accra.

[CIT0015] Golden M, 1988, ‘The effects of malnutrition in the metabolism of children’, *Transactions of the Royal Society of Tropical Medicine and Hygiene* 82(1), 3–6. 10.1016/0035-9203(88)90245-3 3140444

[CIT0016] Hammond T.J, 2014, *Evaluating the knowledge, attitude and practice of rural Guatemalan Healthcare Providers regarding chronic malnutrition in children*, Emory University, Georgia, US.

[CIT0017] Karaolis N., Jackson D., Ashworth A., Sanders D., Sogaula N., McCoy D. et al., 2007, ‘WHO guidelines for severe malnutrition: Are they feasible in rural African hospitals?’, *Archives of Disease in Childhood* 92(3), 198–204. 10.1136/adc.2005.087346 16670119 PMC2083437

[CIT0018] Kgaphola M., Wodarski L. & Garrison M, 1997, ‘Nutrition knowledge of clinic nurses in Lebowa, South Africa: Implications for nutrition services delivery’, *Journal of Human Nutrition and Dietetics* 10(5), 295–303. 10.1046/j.1365-277X.1997.00063.x

[CIT0019] Kim H. & Choue R, 2009, ‘Nurses’ positive attitudes to nutritional management but limited knowledge of nutritional assessment in Korea’, *International Nursing Review* 56(3), 333–339. 10.1111/j.1466-7657.2009.00717.x 19702807

[CIT0020] Kobe J.A, 2006, *Aspects of nutritional knowledge, attitudes and practices of nurses working at the surgical division at the Kenyatta National Hospital, Kenya*, University of Stellenbosch, Stellenbosch.

[CIT0021] Kushner R.F, 1995, ‘Barriers to providing nutrition counseling by physicians: A survey of primary care practitioners’, *Preventive Medicine* 24(6), 546–552. 10.1006/pmed.1995.1087 8610076

[CIT0022] McWhirter J.P. & Pennington C.R, 1994, ‘Incidence and recognition of malnutrition in hospital’, *BMJ* 308(6934), 945–948. 10.1136/bmj.308.6934.945 8173401 PMC2539799

[CIT0023] Mihalynuk T.V., Coombs J.B., Rosenfeld M.E., Scott C.S. & Knopp R.H, 2008, ‘Survey correlations: Proficiency and adequacy of nutrition training of medical students’, *Journal of the American College of Nutrition* 27(1), 59–64. 10.1080/07315724.2008.10719675 18460482

[CIT0024] Mogre V., Ansah G.A., Marfo D.N. & Garti H.A, 2015, ‘Assessing nurses’ knowledge levels in the nutritional management of diabetes’, *International Journal of Africa Nursing Sciences* 3, 40–43. 10.1016/j.ijans.2015.07.003

[CIT0025] Park K., Cho W., Song K., Lee Y., Sung I. & Choi-Kwon S, 2011, ‘Assessment of nurses’ nutritional knowledge regarding therapeutic diet regimens’, *Nurse Education Today* 31(2), 192–197. 10.1016/j.nedt.2010.05.017 20621397

[CIT0026] Schaller C. & James E.L, 2005, ‘The nutritional knowledge of Australian nurses’, *Nurse Education Today* 25(5), 405–4412. 10.1016/j.nedt.2005.04.002 15946775

[CIT0027] Schofield C. & Ashworth A, 1996, ‘Why have mortality rates for severe malnutrition remained so high?’, *Bulletin of the World Health Organization* 74(2), 223. 8706239 PMC2486901

[CIT0028] Schofield C. & Ashworth A, 1997, ‘Severe malnutrition in children: High case-fatality rates can be reduced’, *Africa Health* 19(6), 17–18. 12321237

[CIT0029] Tafese Z. & Shele A, 2015, ‘Knowledge, attitude and practice towards malnutrition among health care workers in Hawassa City, Southern Ethiopia’, *Global Journal of Public Health Research* 1(1), 1–8.

[CIT0030] United Nations Children’s Fund, 2013, *Improving child nutrition: The achievable imperative for global progress*, United Nations Children’s Fund, New York.

[CIT0031] Waitzberg D.L., Caiaffa W.T. & Correia M, 2001, ‘Hospital malnutrition: The Brazilian national survey (IBRANUTRI): A study of 4000 patients’, *Nutrition* 17(7), 573–580. 10.1016/S0899-9007(01)00573-1 11448575

[CIT0032] Waterlow J, 2000, ‘Intensive nursing care of kwashiorkor in Malawi’, *Acta Paediatrica* 89(2), 138–140. 10.1080/080352500750028717 10709879

[CIT0033] Yalcin N., Cihan A., Gundogdu H. & Ocakci A, 2013, ‘Nutrition knowledge level of nurses’, *Health Science Journal* 7(1), 99–108.

